# Socio-economic impact of COVID-19 on rural livelihoods in Mbashe Municipality

**DOI:** 10.4102/jamba.v14i1.1361

**Published:** 2022-10-31

**Authors:** Thandeka Khowa, Anelisiwe Cimi, Tafadzwa Mukasi

**Affiliations:** 1 Department of Sociology, Faculty of Social Science, North-West University, Mafikeng, South Africa; 2 Department of Human Settlements, Faculty of Social Sciences, University of Fort Hare, Alice, South Africa; 3 Department of Development Studies, Faculty of Management and Commerce, University of Fort Hare, Alice, South Africa

**Keywords:** livelihoods, COVID-19, lockdown, pandemic, socioeconomic impact

## Abstract

The study sought to examine the impact of coronavirus disease 2019 on the socioeconomic livelihoods of rural communities by focusing on the Good Hope community under Mbashe Municipality. A qualitative research methodology was employed through in-depth interviews and focus group discussions. The study sought to examine the impact and survival strategies employed by the Good Hope community with the outbreak of the coronavirus, which has widely affected the world since 2019. The study further aimed to examine the role of the South African government in assisting rural communities since the outbreak of the coronavirus.

**Contribution:**

The study’s findings highlight the lack of proper intervention strategies employed by the government in assisting rural communities. Communities including Good Hope have been hard hit by the pandemic and continue to suffer under the implemented lockdown regulations imposed by all governments globally.

## Introduction

The coronavirus disease 2019 (COVID19) is a virus that causes respiratory infections ranging from the common cold to more severe diseases such as Middle East Respiratory Syndrome (MARS) and severe Acute Respiratory Syndrome (SARS) (National Institute for Communicable Diseases 2020). The The COVID-19 has created a state of socioeconomic instability as it brought people’s lives to a standstill and led to the suspension of economic activities (Rogan & Skinner 2019). The COVID-19 was first reported in Wuhan, China, in December 2019, and has continued to wreak havoc worldwide since then. South Africa reported its first case of COVID-19 on 05 March 2020 (World Health Organization 2020). The first reported case in South Africa was of a returning citizen from a holiday in Italy (National Institute for Communicable Diseases 2020). The group arrived on 01 March 2020. On 15 March 2020, the South African government declared a state of national disaster because of the exponential increase of confirmed cases of COVID-19 among the citizens. The COVID-19 has had a devastating impact worldwide; it is imperative to pinpoint that it has crippled the whole globe economically (Fernandes 2020). Its spread has left businesses worldwide counting and wondering how they will recover from the profit lost during the pandemic. Many people have lost their jobs, and others had their income cut because of the pandemic, and unemployment has increased across significant economies.

## Literature review

In the United States of America, the proportion of people out of work has hit 10.4%, signalling an end of an increase for one of the world’s largest economies (International Monetary Fund [IMF] 2020a). International Monetary Fund (2020a) claimed that the global economy would shrink by 3.0%, and it described the decline as the worst since the Great Depression of the 1930s. However, data differ between countries; the global spread of COVID-19 and the rising number of confirmed coronavirus cases in Africa have raised concerns about its weak healthcare systems, while the lockdown has caused economies to contract substantially (South Africa Department of Health 2020). The COVID-19 has forced many countries to enforce lockdown restrictions to prevent escalating infection numbers. South Africa enforced the lockdown restriction on 26 March 2020, and declared a national state of disaster. The lockdown restrictions aim to curb the high rates of infection and ensure a state of readiness for the health sector. South Africa is a developing third-world country with high unemployment rate with many people surviving in the informal sector; thus, the outbreak of COVID-19 has negatively impacted the citizens (Chotiner 2020).

According to Statistics South Africa (2020), the unemployment rate jumped from 23.3% to 30.0%; it increased by 7.5 percentage points in the third-quarter compared with the second-quarter making it the highest unemployment rate recorded since 2008. International Labour Organization (2020a) stated that South Africa is among the many severely infected countries globally; the COVID-19 pandemic hindered the progress made in the last 26 years since the new dawn of democracy. The COVID-19 pandemic erupted when the South African economy was already critically challenged with pedestrian growth and limited wealth redistribution, making South Africa one of the most significant unequal countries in the world. The pandemic has resulted in the loss of jobs and lives and sickness, and has put millions of livelihoods in danger (Adams-Prassl et al. 2020; World Investment Report 2020). The COVID-19 has spread shockingly, contaminating millions of people and getting economic activities to a near dead end as countries enforced strong restrictions on movement to control the spread of the virus (Doerr & Gambacorta 2020).

The COVID-19 pandemic has intensified food insecurity in urban and rural areas because of the disruption in the food supply chain, increased physical and economic barriers that restrict access to food, and the catastrophic increase in food waste because of labour shortages (ILO 2020b). Agriculture has been seen as the survival strategy during this pandemic which plays a vital role in economic development and can contribute significantly to household food security (Dongyu 2020). According to Kiaga, Lapeyere and Marcadent (2020), subsistence farming has been rising in rural areas and is advancing food and nutritional security during the pandemic. Gardening promotes food security and is also a coping mechanism that promotes mental and emotional health during the lockdown. Staying outdoors around plants and nature and doing physical exercise in the garden have helped many people cope with the anxiety that comes with the lockdown. Self-employed people and people working from home are also looking for activities to occupy their free time; parents too are turning to gardening as an outdoor activity with children stuck at home after the schools were shut (Kiaga et al. 2020).

In response to the COVID-19 crisis, governments put large amounts of money into response strategies to ensure that people are surviving during this time. However, not all governments could afford funds for these response strategies, which meant that underdeveloped and developing countries had to borrow more money from the international financial institutions to fight the spread of the pandemic. Practically, half of all low-income nations live with extraordinary debt levels, and have been since before the eruption of COVID-19. Developing countries’ debt in GDP percentage had risen from an average of 33.1% between 2010 and 2016 to 50.1% in 2019 (IMF 2020a).

An African Union study on the economic impact of COVID-19, released in April 2020, indicated that the continent lost up to $500 billion and that African countries may be forced to borrow heavily to survive after the pandemic (IMF 2020a). The lack of fiscal space for African countries to tackle the pandemic and its repercussion attributed to many challenges. According to Hepburn et al. (2020), the first encounter is high debt to unsustainable gross domestic product (GDP) levels. The second challenge is the high economic deficits which mean gaps between spending and revenues have forced countries to explore alternative financing for development projects. Therefore, loans become an alternative but that further worsens their debt burden. The third encounter is the high cost of borrowing with interest charges of between 5% and 16% on 10-year government bonds, compared to near-zero to negative rates in Europe and America (World Economic Situation and Prospects 2020). However, interest repayments constitute the highest payments portion and the fastest-growing budget expenditure in African economies. Lastly, the depreciation of many African currencies against major international currencies has activated inflation. For example, Botswana Pula and the South African Rand have lost approximately 8% of value against the US dollar and the euro since the eruption of the COVID-19 pandemic (Hepburn et al. 2020).

The South African government borrowed about 4.3 bn from the IMF to fight the spread of the COVID-19 pandemic (IMF 2020b). These funds aim to assist South Africans in food parcels, personal protective equipment (PPEs), social relief grants, unemployment insurance fund (UIF), etc. With lockdown restrictions, many people, especially those who rely on the informal sector, could not trade their labour. Thus, for many families living in poverty, the government had to implement strategies for survival; whether these strategies were successful and beneficial has been a source of great debate. On the 23 March 2020, the South African government set up the Solidarity Fund, a special purpose agency to help government and civil society fund South Africa’s response to the COVID-19 pandemic to prevent its further transmission and support those affected (Aldaco, Hoehn & Laso 2020). The Solidarity Fund has received pledges totalling R2.6 bn up to 23 April 2020, with R1.1 bn designated for healthcare-related struggles (Aldaco et al. 2020). The R2.6 bn in pledges from tens of thousands of South Africans has been disbursed by more than R1 bn in critical interventions, such as the procurement of PPEs for frontline health workers and disbursement of food parcels for disadvantaged people, to help South Africa respond to the socioeconomic impacts of the COVID-19 (Pressman, Naidu & Clemens 2020).

From April 2020, the government distributed food parcels to the needy who were hit hard by the lockdown. The food parcels were distributed by the National Departments that allocated R20 million and R23 million from the Solidarity Fund to provide emergency food parcels of R700 per household (Akwa, Ning & Maingi 2020). The Department of Social Development distributed 250 000 food parcels across the country together with non-governmental organisations (NGOs) and community-based organisations (CBOs). These were to ensure that no person goes to bed hungry (Akwa et al. 2020). The South African Social Security Agency (SASSA) issued the food parcels as a temporary provision of assistance for persons in dire need and unable to meet their most basic needs.

In addition, the COVID-19 outbreak led to an increase in demand for PPEs, particularly disposable masks, because the government had instructed people not to leave their homes without wearing a mask (Burki 2020). Kamerow (2020) argued that the demand for PPEs has reached unmatched levels as COVID-19 has spread globally, and governments have sought to prepare and respond. Social grants play an important role in mitigating the effects of poverty for children and their families. Numerous studies have found that receiving social grants is linked to improved nutrition and health outcomes, and protecting families against major financial shocks (Grinspun 2016). South Africa primarily provides grants for people too old to work, the disabled, and children. During the COVID-19 outbreak, people depending on social grants have struggled as the cost of living rose. A project that monitors food prices found that the cost of a low-income household food basket increased substantially over the first 3 weeks of March 2020 as the pandemic unfolded in the country (Pietermaritzburg Economic Justice and Dignity 2020).

The government has responded to the outbreak impact of the COVID-19 pandemic by increasing the amount of all existing social grants for 6 months (Bhorat & Köhler 2020). The government introduced a R500 bn fiscal support package that includes 6 months increase for social grants (Bassier et al. 2020). South African President Cyril Ramaphosa declared an important package of socioeconomic measures to address the consequences of the national lockdown. The supplementation of the grant that is paid to the guardian was increased to R740 per child in May 2020. From June to October 2020, child support grants decreased to their original amount, R440 per month, and guardians received an additional R500 per month. An increase of payment per guardian means that instead of a household with three children receiving an extra R1500 per month, they only get an additional R500, the same amount as a family with one child. For 6 months, all other grants have been increased by R250 per month (Bassier et al. 2020). The increase by R250 and R500 for disaster relief of social grants for 6 months originated to R20 bn and R40 bn, which the government received from commercial bank loans (Bassier et al. 2020).

South Africa has further established the social relief grant as a response strategy of R350 a month. The grant is given to unemployed people who do not receive any form of stipend (Goodell 2020). Up until this day, many people have not received the R350 relief fund because it requires a lot of documentation (Gumede 2020). The provision of knowledge awareness and vaccines has remained a priority, especially in rural communities that are home to many older people of the country, the most endangered from the coronavirus. The uncertainty about the management of COVID-19 has led many countries with poor healthcare to resort to home remedies to treat the virus. Herbal medications are becoming increasingly popular with the general public during COVID-19 (Benarba & Pandiella 2020). Knowledge regarding indigenous medicines is minimal, while the need for such knowledge is becoming more essential. In Africa, approximately 80% of people rely on herbal medicine for primary healthcare as they are poor and have no money for the medication (Mars 2007).

## Theoretical framework

The crisis theory founded by Caplan in 1964 guides this study. the theory is further articulated by Walby, 2021 in his understanding of the covid-19 pandemic. Walby (2021) stated that during a crisis, the person’s functioning becomes disorganised; if the crisis continues, the tension mounts beyond, and the affliction increases over time to a breaking point. This results in a major breakdown in mental and social functioning. Many people encounter some degree of helplessness, anxiety, frustration, inadequacy, and depression.

The works of Caplan, 1964 on crisis theory remain crucial in giving the basis for crisis theory. These were established at a period in the state’s history whereby fast response to large scales of shocking trials such as World War II and other disasters was essential. Caplan articulated that a crisis is precipitated when a person encounters a problem that appears not to have an instant solution. Consequently, a period of upset and tension comes after a person has made several attempts to solve the problem he or she is encountering. Caplan argued that everyone faces various crises in the course of living, and some human beings may or may not be capable of coping with crises. When a person’s coping mechanisms are inadequate to solve the difficulties created by a stressor event, this will lead to anxiety, and the person will be in a state of active crisis.

The crisis theory suggests that every perilous event that produces stress results in a state of active crisis, and there are recognisable situational components. The situational components signify a challenge to be solved by a person who is exposed to the crisis event (Walby 2021). However, the inability to generate a solution to the problems does not exist in mirror pathology itself because environmental factors also contribute to this difficulty. When an individual is in a state of active crisis, he is more emotionally disturbed and is willing to accept change than at any other time. Caplan stated that a crisis is twofold: firstly, a normal crisis, whereby the individual can or cannot cope, and secondly, situational crisis, whereby some people may be able to adapt, while others may not and be overwhelmed. A situational crisis responds to a common situation, such as illness.

The implications of COVID-19 pose new threats as the public grieving for deceased loved ones is threatened by social distancing demands. The pandemic also created new and unfamiliar hazards such as sudden and prolonged closure of schools and abrupt cancellations of certain rites of passage such as graduations and weddings. Human behaviour is crushed mainly by life events that cannot be controlled; at times, the event is far too complicated to be understood by an individual (James & Gilliland 2017). The COVID-19 has caused a social extensive range crisis which has possibly led to quick adjustment to an alternative practice of society; it has changed what was known to be functional by the Good Hope location. In addition, unique elements of the COVID-19 pandemic increase the risk for crisis; however, crisis theory seeks to balance the need to meet basic needs and earn a livelihood with mitigating exposure to illness and the spread of COVID-19.

## Methodology

A qualitative research design was adopted for the study using in-depth interviews and focus group discussions (FGDs). Using purposive sampling, the study selected 20 households in the Good Hope community. Among the people interviewed, there were two government officials: the ward councillor and an official from Mbhashe Municipality. These officials were selected because they are responsible for implementation of services in Good Hope community. The participants provided more information on the role played by the government in assisting the people of Good Hope location. In-depth interviews and FGDs were used to collect data for the study. In-depth interviews were carried out with the heads of the households to understand the socioeconomic impact of COVID-19 on their families because they are the ones that provide for their families. In the FGDs, two groups had five people in each group, and they were carried out for 45 min. Morris (2015) stated that for issues of concentration levels, in-depth interviews should not exceed 45 min.

These groups included other members of the households that did not have a chance to participate in the in-depth interviews, as they also provided different views on how COVID-19 has affected them. The study was guided by the ethical standards of the University of Fort Hare.

### Ethical considerations

Approval to conduct the study was obtained from the Faculty of Humanities and Social Sciences Research Ethics Review Committee, University of Fort Hare. No reference number was assigned at this time.

## Findings

### Socioeconomic impact

#### Unemployment

Economically, COVID-19 profoundly impacted people’s lives in Good Hope. Within the COVID-19 pandemic most employed people lost their jobs, resulting in their families being affected by poverty. Many households suffered financially through the death of breadwinners. In some instances, people were temporarily forced to stop work and stay at home without a salary. In Good Hope, people experienced significant shocks to their livelihoods, and the threat of hunger presented a substantial concern for health and social stability. Most people are struggling to rebuild and recover economically because of the loss of employment, and large groups of people live in hunger. One of the participants from the study said:

‘I lost my job at work they had to retrench a lot of workers because the company had no funds to operate due to COVID-19 national lockdown. I had to borrow money from a loan shark in order to buy groceries to feed my family during level 5 of the national lockdown, a loan that I do not know how I am going to pay back since I am currently unemployed.’ (FGD 1, Participant 5, 2021)

From the aforementioned information, it is clear that unemployment resulted in many people being victims of debts for survival in Good Hope. This puts citizens under more financial pressure as they will have no means of paying back. According to the World Investment Report (2020), the COVID-19 pandemic has resulted in the loss of jobs and has put millions of livelihoods in danger; this is evident when wage earners lose their jobs, fall ill or die. In Good Hope, many people lost their jobs, and in some cases, breadwinners died from the virus. Good Hope people were also the victims of retrenchment because of the impact of the COVID-19 pandemic. This reveals that one of the organs of society, the economy, has been affected due to COVID-19 leading to the economic instability and disruption of the normal functioning of the society. This is in line with the crisis theory, which argues that when one part of the social institutions is dysfunctional, it affects all the other parts and creates social problems, prompting social change (Walby 2021).

#### Closure of small businesses sector

The government’s restrictions to curb the spread of COVID-19 forced the self-employed (the street vendors, hairdressers) to close their businesses. These businesses include: salon owners, hawkers and small businesses, whose livelihoods depend on daily interactions with the public. In Good Hope, self-employed workers have experienced significant declines in income and working hours during the COVID-19 crisis. Some of these people are in distress as they do not qualify for the unemployment benefits, such as the social relief grant and the UIF. Moreover, they often do not have sufficient savings for survival without an income:

‘The outbreak of COVID-19 has affected my business…, we were not allowed to sell in town as I am a street vendor, I had to stay at home and adhere to lockdown regulations, especially during level 5 of the lockdown. It was difficult to have an income as my business was the only source of income for me.’ (in-depth interview, participant 5, 2021)

From the previous findings, it is clear that the lockdown that was introduced by the government to fight the spread of COVID-19 has severely impacted the informal sector. The findings reveal the economic struggle that the informal workers were subjected to due to the lockdown, for instance, closure of their small businesses as per the regulations of lockdown. This is in line with Bartik, Marianne and Zoe (2020), who argued that the informal sector was affected by the COVID-19 lockdown, restrictions on people’s movement and closure of non-essential service businesses.

#### Domestic violence and gender-based violence

Loss of jobs led to the rise in domestic violence because of the stresses caused by the economic hardship faced during this time. The anger and pain that people experienced, they took it out by physically, financially and emotionally abusing their partners and other family members:

My husband lost his job in April 2020 and after that there was no peace in our home ….he always shouted at them (kids) without a valid reason. …for the first time in 15 years of marriage he slapped me in front of of the kids.’ (In-depth interview, participant 1, 2021).

The earlier findings disclose that the loss of jobs and socioeconomic impact of COVID-19 resulted in family hardships and increased the rate of gender-based violence in many households in the study area. Haneef and Kalyanpur (2020) claimed that the stay-at-home measures led to an unexpected decline in household income because of job losses. Thus, making abuse survivors more dependent on their abusers, and the accompanying stress and hardships, together with close contact with the abuser, often provoke more violence.

#### Closure of educational institutions

In line with the government’s response to COVID-19, all the schools in Good Hope were closed down to save lives. However, private schools were operating, but children were not allowed to attend as per the regulations of lockdown. The shutting down of schools has had an economic and social impact on parents. Closing schools meant that parents had to dig deep into their pockets to feed their children while they were indoors during the lockdown period. Private schools gave children and parents tasks for home-schooling, but it was a challenge as some parents were illiterate and did not have time to help with schoolwork.

On the other hand, the lockdown has shifted away from contact classes to online classes, affecting university students residing in Good Hope. Online learning introduced as a way of teaching and learning posed many challenges to the students in Good Hope. Online learning requires not only electricity but also devices through which the online learning information and materials can be accessed. Students from the Good Hope location do not have access to these technological devices (laptops and computers) or network coverage and lack basic digital skills. To attend virtual classes, one needs to have a certain degree of technical skill, including successfully logging in, contributing to classes, submitting work, communicating with teachers and understanding online communication. Good Hope is not technologically advanced, and the shift away to online learning took the village by surprise. Some students could not participate in online classes since they live in overcrowded houses, thus making it difficult for them to pay attention to classes and do school work:

‘…I do not know how to use Blackboard, Zoom and other apps… I do not know how to submit my assignments and there is no one who can assist me at home… …I do not know how to log in.’ (in-depth interview, participant 14, 2021)

According to Brooks et al. (2020), the closure of schools in South Africa has harmed disadvantaged students and deprived them of opportunities for growth and development. The online learning challenges such as network unavailability, lack of access to technology, and residing in overcrowded areas further exclude Good Hope students from accessing online education. The study area requires help from the education sector not to neglect rural students but to equip students who live in rural areas with necessary skills such as digital skills to have equal access to education as those who live in urban areas.

#### Working from home strategy

The lockdown and its restrictions, such as social distancing and quarantine, led to many businesses implementing the working from home or remote working strategy. In Good Hope, employed residents had to adopt the working from home strategy to protect themselves from the COVID-19 infections. Some employees experienced unavailability of network coverages and the challenge of power outages caused by broken electricity cables around Good Hope. On the other hand, for some employees, it was beneficial as they got an opportunity to spend time and take care of their families:

‘… I enjoyed working at home because it has given me enough time to spend with my kids…, on the other hand it helps me in saving financially since I had a helper who took care of them before the lockdown….’ (in-depth interview, participant 17, 2021)

‘In my house I do not have electricity as my house is new… I am not used to technology, so it was hard for me to adapt to the use of Microsoft things, although we were taken through some training about how to use it, I still struggle….’ (In-depth interview, participant 18, 2021)

The responses from participants made it clear that the working from home strategy, which many businesses have adopted, has been problematic for the majority of employees in Good Hope. This occurred on account of several issues, including connectivity issues, power issues, non-conducive places to work and household chores. Consequently, Fairlie (2020) argued that the disadvantage of working from home is the loss of productivity because of the distraction of comfort of taking unnecessary breaks during work. Another issue is the lack of network coverage required to perform the work from home strategy, as many underdeveloped and developing countries lack quality network coverage (Fairlie 2020). In developing and underdeveloped countries, many people reside in deep rural areas where there is no power, and this has been the case for the employed residents of the Good Hope location.

#### Survival strategies during coronavirus disease 2019

Businesses in the informal sector have suffered a significant loss during the outbreak of COVID-19 and lockdown. In Good Hope, the informal sector includes street vendors, spaza shop owners, hairdressers and taxi drivers. All informal workers like street vendors whose livelihoods depend on being in public spaces to sell their products have been hit hard by the lockdown because of the closure of businesses to adhere to the regulations imposed by the government. As a response to the disturbances made by the pandemic and the lockdown imposed, people had to come up with ways of surviving during the lockdown:

‘As a way to survive I decided to do a vegetable garden so that we could eat at home and sell other veggies to the neighbours. The garden helped a lot because I was able to buy other things from the money I made from selling to people in the village.’ (FGD 1, participant 1, 2021)

‘When I closed down the salon, I decided to do house calls for people who live in the village and nearby villages. I ensured that only one person came a day to avoid overcrowding and I made sure that my clients wore masks.’ (In-depth interview, participant 16, 2021)

‘What helped me to survive was my Forever Living business because I was able to run it on Facebook, and orders were coming in for the immune system boosters because of the virus we are facing.’ (in-depth interview, participant 3, 2021)

Several informal workers faced some challenges in feeding their families during the lockdown. As a result, they came up with strategies to survive, including traditional ways to provide food in rural areas, such as subsistence farming. This proves what Kiaga et al. (2020) said about the rise in the practice of subsistence farming in rural areas during the COVID-19 pandemic. They gained food and income from subsistence farming while their businesses were still closed. Other residents survived by running different businesses that did not break the ‘stay-at-home’ measure introduced.

From the findings mentioned earlier, it is clear that people who solely depended on informal businesses for survival were severely impacted by the COVID-19 pandemic and struggled to provide for their families. However, some of these people got relief from the established government strategies, such as the Social Relief of Distress (SRD) grants.

#### Unemployment insurance fund

The crisis theory claims that when people are in a state of crisis, are anxious and open to help. Therefore, the logic for crisis theory is the belief that providing support and guidance to people in crisis will provide sustained mental health problems. Caplan (1964) defined a crisis as a threat to homeostasis; in a crisis, an imbalance occurs that results in confusion and disorganisation. In Good Hope, there has been a delay on UIF, which has affected the people; this has forced people to sell their livestock to survive. Some employers caused UIF claims to delay by not submitting the UIF application forms. Moreover, corruption by some companies delayed the process, whereby the employers claimed money on behalf of their employees but did not disburse it to them:

‘I was not working since the lockdown was implemented and it has been very hard for my family and me. I expected that I would get the assistance of UIF. I applied for it but did not get it; when I asked my employer, he did not give me a straight response. I was very frustrated, then I decided to go to department of labour where I found out that the payment for the UIF had been made to the company, but my employer denied that, so I ended up not getting it.’ (In-depth interview, participant 16, 2021)

The previous finding indicates that households in Good Hope where formal sector jobs have been lost are struggling financially and suffering from hunger because of the corruption and delays in receiving their UIF payments. The South African Broadcasting Corporation (SABC) (2020) reviewed that there were circumstances where most of the time, employers would apply for 20 employees. However, in the database, there were only 10 employees that have been declared. There were also instances where employers received funds to pay employees’ UIF, but these funds did not reach the employees, or some overclaims that did not match the existing staff or the period. Accordingly, this proves that a lot of corruption has been happening concerning the payments of UIF.

#### Social relief of distress grant

The government introduced a special COVID-19 SRD of R350 a month to unemployed individuals. In line with the arguments of the crisis theory, an individual in crisis experiences the intensified need for help, and the signs of distress induce a helping response from those around; therefore, the SRD grant was the government’s response strategy for helping people that are financially affected by the pandemic. From the households that were interviewed, majority reported that they did not receive social relief grants due to a lack of knowledge of the grant and documentation required for the grant application. Moreover, people have to stand in long queues, and post office workers need bribes to give people their money; as a result, many people do not collect their money. Issues of social distancing at pay point have been reported as a push factor prohibiting people from collecting the grant because of fears of getting infected:

‘…I did not get the grant because I had no money to bribe the people that work at the post office, many people bribe in order for their process of getting money to be fast-tracked.’ (In-depth interview, participant 5, 2021)

‘I did not get the money because my village is far from town and there are long queues in the post office people have to arrive there at 4 a.m. another thing I do not even have money to take a taxi.’ (In-depth interview, participant 7, 2021)

The findings agree with Gumede (2020) in that many people have not received the R350 relief fund because of the application process. Based on the previous results, only a few Good Hope occupants got their social relief grant. Bribery and residing in disadvantaged areas are the key stumbling blocks to getting social relief grants.

#### Vaccine

The introduction of the COVID-19 vaccine has been a relief as it is anticipated that it will reduce the number of infections. Crisis theory argues that a crisis can have harmful effects unless successfully dealt with. Furthermore, the theory indicates that there should be an action to restrain the crisis. The South African government has provided the COVID-19 vaccine as a response strategy for the virus. However, there has been a lot of misinformation about the vaccine and its development. There is an urgent need to educate people about the COVID-19 vaccines, including Good Hope residents, to correct the misinformation spreading in societies and on social media. The traditional leaders and councillors in Good Hope have taken the responsibility to educate everyone about COVID-19 through awareness campaigns. However, the awareness campaigns have not educated people about the COVID-19 vaccine, and as a result, many rural residents were hesitant to be vaccinated due to misinformation:

‘…I heard that it can change a person’s DNA, so what will I do if it changes my children’s DNA and their father later wants a DNA test? It has been also said that the vaccine causes infertility….’ (In-depth interview, participant 11, 2021)

‘I am never taking the vaccine what if the government wants to reduce the number of people in the country by injecting us with the virus.’ (In-depth interview, participant 1, 2021)

The findings have shown inadequate knowledge about the COVID-19 vaccine; thus, the government needs to initiate more health education programmes for the vaccine rollout in Good Hope. The residents are far behind in terms of vaccination knowledge, and a lot still needs to be done through awareness programmes.

## Conclusion

This qualitative study examined the impact of COVID-19 on the socio-economic livelihoods of Good Hope people in Mbashe Municipality. The study findings revealed that the Good Hope community were impacted exceptionally due to the implementation of lockdown to curb the rate at which the global pandemic is spreading. Furthermore, findings of the study highlighted dire economic struggles people are subjected to. Many people lost their jobs, and some closed their businesses in line with lockdown regulations which intensified the economic conditions. In addition, the outbreak of COVID-19 has also affected people socially because of the stay-at-home measure that has imposed gender-based violence. The closure of schools and the shift to online learning affected the education sector. Moreover, the loss of freedom and separation from loved ones imposed by lockdown regulations led to many people experiencing mental issues such as anxiety and depression. From the study findings, there is a need for the government to improve the health systems and curb corruption.

## Recommendations of the study

The South African government should invest in healthcare infrastructure and take lessons from the outbreak of the previous diseases even though the nature of the diseases differs. The government should also build tax capacity so that it could help people, especially the disadvantaged, because most of them are severely impacted during pandemics. Furthermore, the government can provide the SASSA R350 in the form of a voucher which can be redeemed or withdrawn at any local retail store. Mbhashe Municipality should provide the community members with seedlings for subsistence farming, thus avoiding dependence on the government.

Furthermore, the government should introduce legislation to protect citizens’ livelihoods and help reboot the economy after the pandemic, thereby mitigating unemployment risks during crises and sustaining families’ income. Moreover, a new legislation on social welfare was introducded to support disadvantaged people who may not survive the economic crisis during a pandemic. The government should assist the underprivileged that are deprived of basic subsistence during this crisis through electronic food vouchers to reduce the risk of contracting the virus.
